# Investigation of the Mechanical Behavior of Acacia—Raffia Natural Fiber Composite

**DOI:** 10.3390/polym15153249

**Published:** 2023-07-30

**Authors:** Karthick P, Bindu Madhavan Vijaya Ramnath, K. Palanikumar

**Affiliations:** 1 Department of Mechanical Engineering, Anna University, Chennai 600025, India; 2 Department of Mechanical Engineering, Sri Sai Ram Engineering College, Chennai 600044, India; 3 Department of Mechanical Engineering, Sri Sai Ram Institute of Technology, Chennai 600044, India

**Keywords:** acacia fiber, raffia fiber, tensile strength, flexural strength, impact, double shear, delamination, morphological analysis

## Abstract

Nowadays, industries place a strong emphasis on low-cost, biodegradable materials with long lifespans. As a result, businesses are concentrating on creating composite materials utilizing the world’s plentiful supply of natural fibers. In this study, acacia and raffia fibers are combined with epoxy resin and a hand layup method to create a biodegradable composite laminate. This article investigates the effect of fiber orientation on the mechanical and morphological evaluation of composite materials that have been manufactured. Three different kinds of composites were fabricated in this work: Composite 1, which contained acacia fiber; Composite 2, which was built of acacia and raffia fiber; and Composite 3, which was made of raffia fiber. While Composite 2 is a hybrid composite in this instance, Composites 1 and 3 are monofiber composites. In accordance with the ASTM standards, testing was performed to investigate the different mechanical behaviors, including tensile, flexural, double shear, delamination, hardness, and impact. The results demonstrate that Composite 1 has strong tensile strength, flexural strength, double shear, and hardness tests with a 45° fiber orientation. The 90° fiber orientation of Composite 1 performs well in the inter delamination test. The result demonstrates that composite 1 of type 0 absorbs greater energy. Additionally, Scanning electron microscopy was used to conduct morphological examinations in order to investigate the internal structural failure of the composites. It was found that the composite laminate has fiber cracks, pullouts, and voids, which were reduced with the right curing times and stress.

## 1. Introduction

Composite materials can replace conventional materials with their superior properties. However, studies of fiber orientation on mechanical behavior are rare because the fabrication of composite laminate with different fiber orientations needs a lot of time and effort. Previous research work with different fiber orientations has been discussed here. Ref. [1], have analyzed the effect of fiber orientation on mechanical properties and machinability of GFRP composites with end milling. It was found that fiber orientation influences the cutting force of composites. Additionally, they found that feed rate influences the machinability index by 50%. Ref. [2], investigated the utilization of natural fibers rather than synthetic alternatives that cannot disintegrate, in order to build green composites. The primary focus of the discussion consisted of Acacia tortilis extraction and characterization as a natural fiber for green composites. The productive results demonstrate Acacia tortilis fibers’ potential for use in green composites. The study by [3], examined the physicochemical composition and fiber characteristics of bark fibers from Acacia leucophloea. The bark fibers had a crystallinity index of 51%, a density of 1385 kg/m^3^, and a cellulose content of 68.09 weight percent. Tensile strength varied from 317 MPa to 1608 MPa, while the Young’s modulus ranged from 8.41 GPa to 69.61 GPa. Thermal analysis revealed that the degradation process has a kinetic activation energy of 73.1 kJ/mol and it takes place at a temperature of 220 °C. These findings point to the possible use of thin reinforcements for composites made from Acacia leucophloea fibers. Ref. [4], have investigated three chemical treatments to improve the mechanical properties and moisture absorption of Luffa sponge (LS) fiber bundles, 10% NaOH and 20% CH_3_COOH, 1.6% CO (NH_2_), 5% NaOH and 5% H_2_O_2_. According to the data, the 10% NaOH and 20% CH3COOH treatment enhanced the tensile strength by 121.3%. However, the 5% NaOH and 5% H_2_O_2_ treatment resulted in a 75.0% loss in tensile strength. Moisture regain is reduced by 29.0%, 16.9%, and 12.4%, respectively, whereas moisture absorption was reduced by all treatments. Ref. [5], have examined the natural fiber composites of abaca and raffia fibers. These composites offer superior tensile and impact characteristics compared to the composites constructed from individual fibers. Failure morphology is characterized using SEM, and manufacturing is carried out using the hand layup method. Ref. [6], have examined biomaterials and their sustainability in engineering and building industry applications. In this paper [7], have evaluated the physicomechanical characteristics of raffia textile fiber. It has a layered structure with a tile and honeycomb-like design. Cellulose, the main component of the fiber, has a 64% crystallinity index. Tensile testing reveals that Young’s modulus is 30 GPa, tensile strength is 500 MPa, and total elongation is 2–4%. Raffia textilis has the best specific mechanical properties of the tested vegetable fibers with a density of 0.75 to 0.07. In this work [8], have evaluated the seasonal fluctuations and phytochemical elements of three different acacia species (which focuses on seasonal changes). Rutin in leaves and catechin in bark are found by using LC–MS analysis. As COX-1 and COX-2 are inhibited, TPC levels are also altered. Rutin concentrations peaked in the winter for A. tortilis and A. longifolia. The study of leaf samples indicates the categorization of the species. Ref. [9], have investigated the effect of fiber hybridization and orientation on the mechanical performance of biofiber composites. Neem, abaca, and glass fibers are used to make three distinct kinds of composite materials in a variety of fiber arrangements. Composites with a 45° inclination and a blend of abaca and neem fibers have the best mechanical properties. Scanning Electron Microscopy was used for the morphological characterization of the composites. Ref. [10], have examined the effect of alkaline treatment on laminated glass and raffia fiber hybrid composites. Alkaline treatment enhances mechanical properties, improves adhesion, and reduces heat conductivity. The findings suggest that treated raffia fibers are used in composites that are reinforced with synthetic fibers. Acacia tortilis is examined by [2], as a viable natural fiber for eco-friendly composites. This emphasizes the availability of eco-friendly alternatives to synthetic fibers that are not biodegradable, such as green fiber-reinforced composites. With promising results, the research focuses on mechanical testing, characterization, and extraction of Acacia tortilis fiber. The development of green composite applications benefits greatly from the use of this fiber. The mechanical properties and water absorption of Acacia Arabica bark fiber composites were studied by [11]. The alkali treatment on the fiber surface, as well as the variable fiber volume percentage and length were investigated by them. The results show that fibers that have undergone chemical treatment have superior mechanical properties and decreased water absorption. The composition with the best characteristics has fiber lengths of 30 mm and a volume percentage of 25%. Scanning electron microscopy revealed the fiber pull-outs in the shattered specimens. Ref. [12], have investigated the chemical structure and heat degradation of acacia planifrons fibers subjected to alkali treatment. Various quantities of alkali were applied to the fibers to improve the crystallinity index and thermal stability. The 5% alkali-treated fibers had the greatest crystallinity index and thermal stability, making them the best choice for reinforcement in polymer composites. Ref. [13], have studied the viscoelastic behavior of Raffia vinifera fibers to enhance bio-composites. The eight-element Burger model provides the best match to the data using the uniaxial tensile creep-recovery test apparatus. The Schapery model is used to study creep-recovery behavior. According to the research, Raffia vinifera fibers are capable of dissipating energy under cyclic loading and repeated step loading. Ref. [14], have evaluated the polymer composites’ alkali treatment and raffia fiber length and their ANOVA approach revealed that the composite’s Young’s modulus increases in value when it is subjected to alkali treatment. The composite with the longer length of 15-mm raffia fiber, achieved the best results. The results of the SEM investigation show that the treated and untreated fibers are identical. Ref. [15], have examined the characteristics of raffia palm fiber and they have found that, the tensile strength is lower than that of other fibers such as hemp, sisal, jute, and flax, but it still yields excellent results in terms of chemical, thermal, physical, and elongation qualities. Ref. [16], investigated the physicochemical properties of alkali-treated Acacia pennata fiber and found that treated fibers had a higher crystallinity index (54.65%) and better chemical characteristics. Ref. [17], examined the mechanical properties of Acacia fibers’ tear strength and found that it was comparatively better than the tensile strength. They also concluded that the adhesion between the fiber and rubber improves the result. Ref. [18], showed the physical, mechanical, and moisture absorption properties of Acacia concinna seed powder and Vachellia seed powder reinforced with short Turkish hemp and epoxy resin in their study by producing several matrixes of different weight percentages of seed powders. They observed that the weight percentage of 7.5 showed that the physicomechanical qualities had improved. Furthermore, the filler ingredient boosts the composites’ binding strength. These fibers are ideal for marine and automotive applications due to their superior moisture resistance. According to the study on the characterization of Acacia Caesia bark fibers performed by [19], the fibers easy splitting and high roughness can be used as filler material to remove the loose areas of the fiber. He also found composite thermal degradation around 308 °C, which has great potential for composite applications. Ref. [20], investigated the physical and mechanical properties of several composites in this study. They treated the fiber with NaOH, sodium bicarbonate, and silane to lower its water uptake. They proposed that, even though synthetic fiber has many advantages, it affects the environment and increases CO_2_ emissions. Hence, they suggested that the future use of nanoparticles as filler materials in natural textiles would reduce pollution. Ref. [21], performed a chemical treatment of alkalization and mercerization in their study. As a result, the interfacial strength between the fibers improves, and fiber interlocking produces excellent mechanical characteristics. This research examined the mechanical properties of natural and synthetic fibers. Ref. [22], developed many compositions of hybrid fibers such as Kenaf glass and flax basalts epoxy, glass flax epoxy, and carbon jute epoxy to find better mechanical characteristics. They concluded that hybrid composites are equivalent to synthetic fibers and have better behavior.

It is clear from the review above that, there has not been much study performed with composites made of raffia and acacia fibers. Furthermore, failure morphological analysis has not been performed on the aforementioned laminate made of fiber. Thus, the goal of the research is to create Acacia and Raffia fiber composites that are both mono fiber and hybrid fiber composites, as well as to analyze their mechanical behaviors and failure morphological analysis.

## 2. Experimental Details

### 2.1. Materials Used

#### 2.1.1. Acacia Fiber

**Figure 1:**
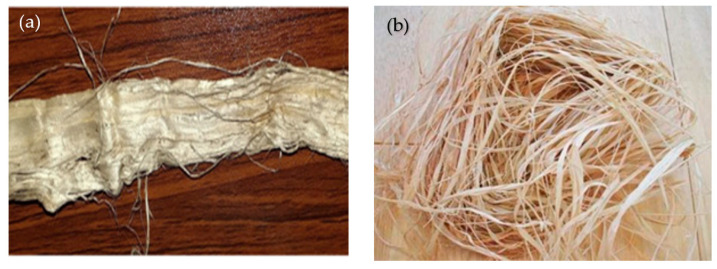
(**a**) Acacia Fiber; (**b**) Raffia Fiber.

Acacia fiber is created by processing acacia gum, which comes naturally from the acacia tree. It is the ideal material for heavy-use items such as dining tables and dining benches because of its density, toughness, and durability. Additionally, it is extremely resistant to fungus, scratch-resistant, and water-resistant, making it appropriate for maritime use. Acacia and raffia’s natural fibers are seen in Figure 1.

#### 2.1.2. Raffia Fiber

Raffia is a natural fiber with characteristics similar to those of jute, bamboo, and hemp fibers. It is made by peeling the leaves from the raffia palm. It offers excellent durability, compressive strength, and other qualities, including strong heat and flame resistance and corrosion resistance. The mechanical characteristics of acacia and raffia fibers are shown in Table 1.

#### 2.1.3. Resin and Hardener

In this study, epoxy resin was employed to fabricate composite laminates, because it has excellent binding capabilities between the fiber layers that create the matrix. LY 556 is an epoxy resin that is used at room temperature. Hardener (HY 951) is used to increase interfacial adhesion and give the composite more strength. In this study, the ideal ratio is selected to get the best outcomes. Table 2 lists the several kinds of composite laminate that were created. Here, type 0 refers to “0” degree orientation, type 45 refers to “45” degree orientation and type 90 refers to “90” degree orientation which is common to all samples in all types of test and are shown in Table 2 below:

### 2.2. Fabrication Process

**Figure 2:**
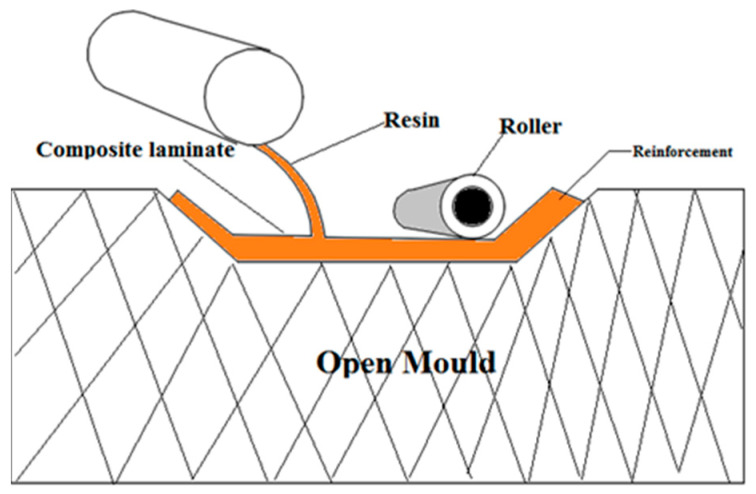
Hand layup process.

**Figure 3:**
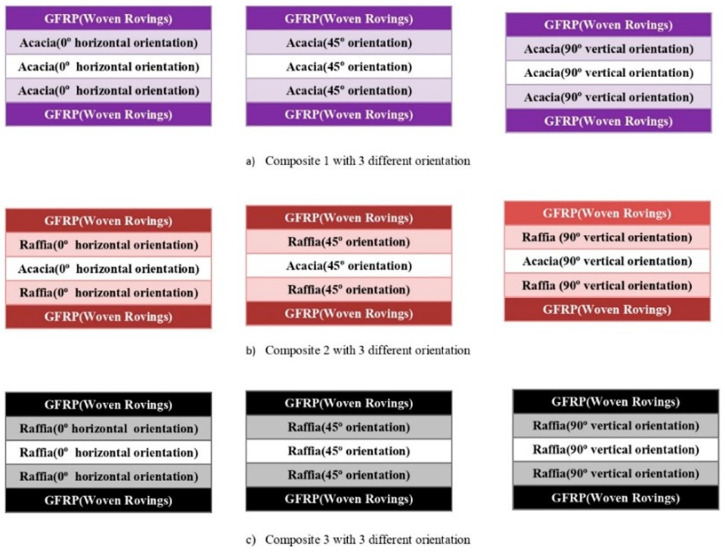
Fiber arrangement of composites’ fabrication.

As illustrated in Figure 2, the composite samples were created by using the hand layup method, which is often preferred and is suited for natural fibers. The fibers are first dried in the sun to eliminate any moisture. The sample specimen’s top and bottom layers are constructed of GFRP, while its middle layer, depending on the kind of composite used, may be formed of acacia, raffia, or both. After producing the composite laminate, polyvinyl alcohol is used as a releasing agent over a level steel table to make it simple to remove the laminate from the surface. The top and bottom layers of the laminate are first created by rolling three layers of woven roving that have been entirely filled with epoxy glue to release any trapped air. The top and bottom layer constructions are cured for 10–12 h in order to get the appropriate strength. In this study, three distinct fiber orientations of 0°, 45°, and 90° with regard to each layer, are used to create three different kinds of composites, referred to as Composite 1, Composite 2, and Composite 3. Acacia is present in Composites 1, Acacia and Raffia are present in Composite 2, and Raffia Fiber is present in Composite 3. While Composite 2 is a hybrid fiber, Composites 1 and 3 are monofiber composites. Here, Composite 1 is made up of two layers of GFRP laminate on the top and bottom and three layers of Acacia in the center. A total of three layers of acacia, raffia fibers alternately in the center and top, and GFRP laminate in the bottom outermost layer made up Composite 2. Three layers of raffia fiber make up the center layer of Composite 3, while GFRP laminate makes up the top and bottom layers. Three samples of each kind are made, each with an alternate layer and fiber orientation of 0°, 45°, and 90°. Figure 3 shows the layer configurations and fiber orientation.

### 2.3. Testing of Composite

**Figure 4:**
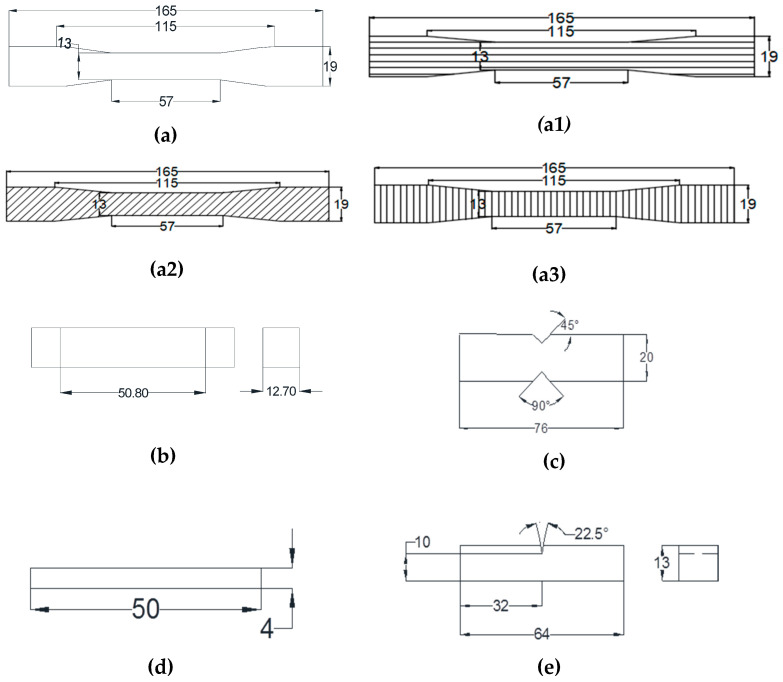
(**a**) Tensile test (**a1**) Tensile specimen for 0° orientation (**a2**) Tensile specimen for 45° orientation (**a3**) Tensile specimen for 90° orientation. (**b**) Flexural test (**c**) Double shear test (**d**) Delamination test (**e**) Impact test.

As per ASTM standards, the specimens are made to examine the mechanical behavior of composite materials. Flexural, tensile, double shear, delamination, impact, and hardness tests of mechanical behavior were performed. Three specimens from each sample were tested, and the average value was used to analyze the results. The test specimens were prepared (see Figure 4) based on ASTM standards for the following tests: the tensile test ASTM D638 (Figure 4a), the flexural test ASTM D790 (Figure 4b), the double shear test ASTM D5379 (Figure 4c), the delamination test ASTM D5528 (Figure 4d), and the impact test ASTM D5947 (Figure 4e). Figure 4(a1–a3) show samples of tensile test specimens with different fiber orientations.

## 3. Results and Discussion

The outcomes of mechanical behavior tests are extensively investigated in this section. The mechanical properties of samples prepared with various fiber orientations have been tested for tensile, bending, double shear, delamination, impact, and hardness.

### 3.1. Results of Tensile Tests

**Figure 5:**
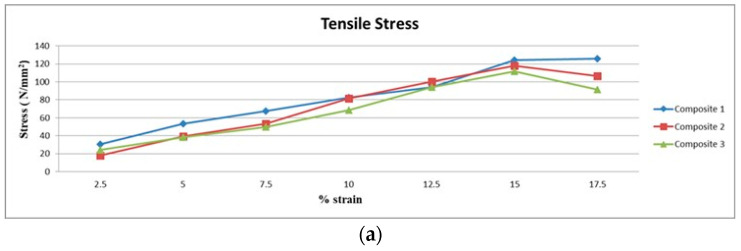

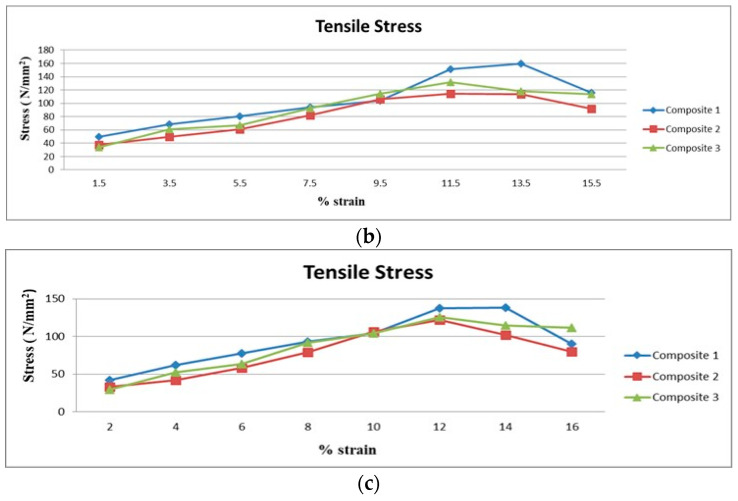
(**a**) Tensile test of 0° orientation in Composites 1, 2 and 3. (**b**) Tensile test of 45° orientation in Composite 1, 2 and 3. (**c**) Tensile test of 90° orientation in Composite 1, 2 and 3.

Figure 5b shows that Composite 1 of type 45 fiber orientation has a very high tensile strength. This is mainly due to the presence of Acacia fiber that, has a high tensile strength, and also fibers aligned at a 45° angle that lock into each other. Figure 5c shows that, Composite 1 with a 90° fiber orientation has the second-best tensile strength among the other composites. This is because, acacia fiber has a high tensile strength and it is also oriented in a way that gives fiber surfaces more surface area to contact.

The type 45 Composite 3 shows the third-best number. When considering tensile properties based on the arrangements of the fibers, type 45 has better tensile strength than type 90. This is mostly because the surfaces of the type 45 fiber that are grouped in different layers of laminate touch each other. This makes the contact area between the fibers larger. The above direction makes the fibers’ load-bearing surface bigger, which makes them behave better mechanically. The resistance surface goes up when type 90 to type 0 are compared in this process.

### 3.2. Result of Flexural Test

**Figure 6:**
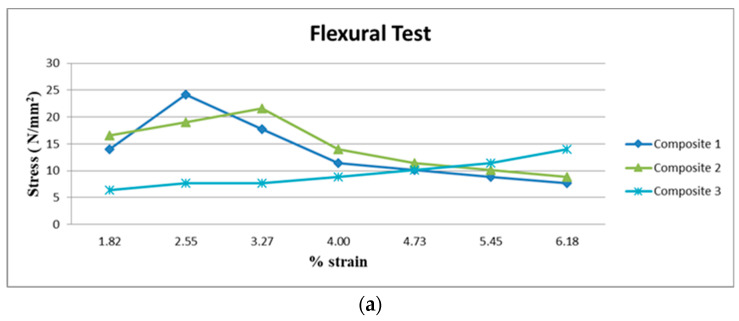

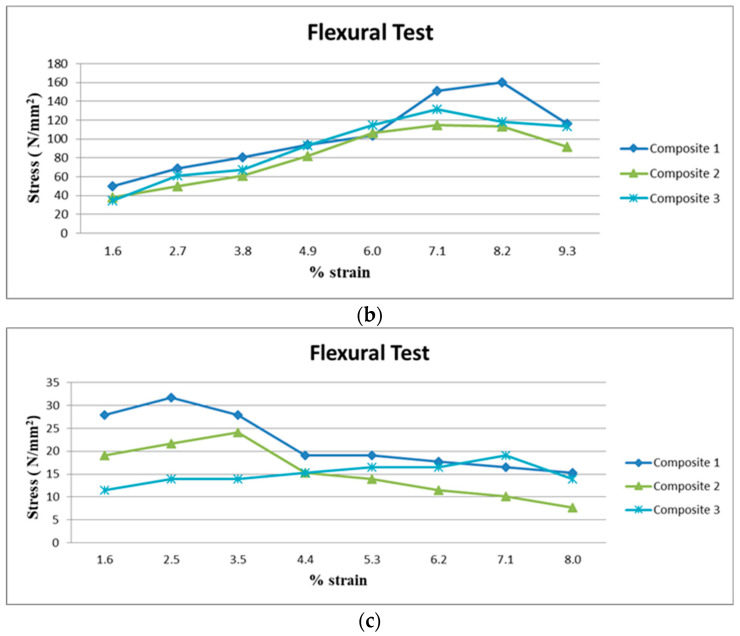
(**a**) Flexural test of 0° orientation in Composites 1, 2 and 3. (**b**) Flexural test of 45° orientation in Composites 1, 2 and 3. (**c**) Flexural test of 90° orientation in Composites 1, 2 and 3.

Figure 6a shows that Composite 1 with a type 45 fiber orientation laminate has a high flexural strength and is followed by Composite 2 with a type 45 fiber orientation laminate. Due to the fact that the Composite 1 laminate comprises acacia fiber, it has a higher strength, with bending resistance. Type 45 also enhances the contact surface, by increasing the surface area in both composites in order to withstand the bending loads. Composite 2 is a hybrid laminate, and the hybrid behavior enhances the type 45 composite flexural capabilities. Because of the poor bending strength and behavior of the raffia fiber as well as the very low surface resistance provided by type 45, Composite 3 of type 45 demonstrates very little flexural strength as shown in Figure 6b.

It was discovered that type 45 exhibits superior flexural behavior compared to the other two orientations with regard to fiber orientation. Additionally, the fibers’ interweaving properties strengthen the composite material resistance to bending. Type 45 has an extremely poor flexural strength and behavior, as a result of the contact surface’s inability to effectively withstand bending loads.

### 3.3. Result of Double Shear Test

**Figure 7:**
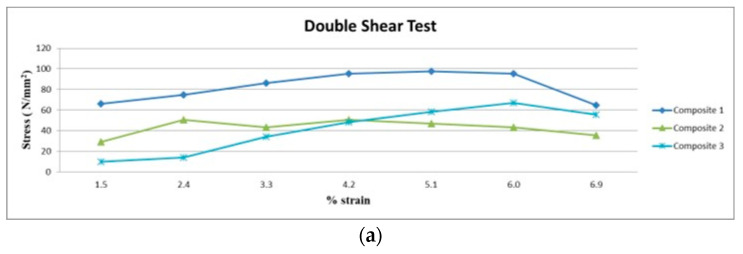

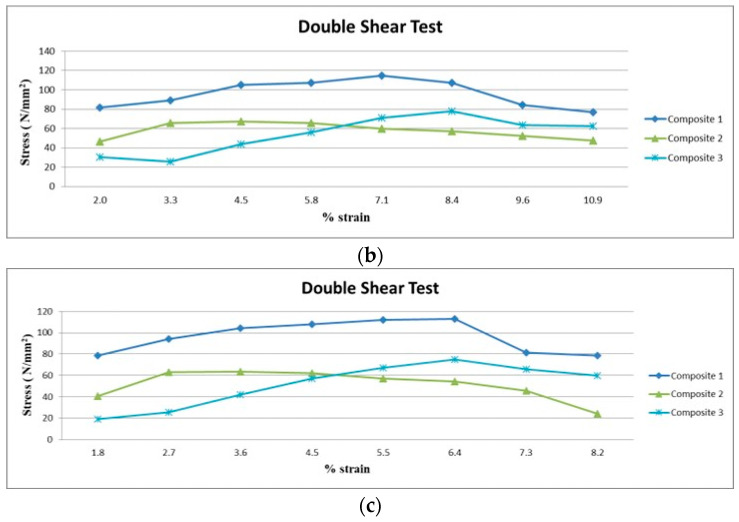
(**a**) Double shear test of 0° orientation in Composites 1, 2 and 3. (**b**) Double shear test of 45° orientation in Composite 1, 2 and 3. (**c**) Double shear test of 90° orientation in Composite 1, 2 and 3.

Figure 7a–c shows the shear behavior of composites. Comparing Composite 1 of type 45 to other samples, it exhibits superior double shear behavior. Composite 1 of type 90 comes next. Type 45 exhibits improved mechanical behavior, because it provides the strongest resistance to fracture initiation in the fiber laminate. Additionally, the acacia fiber has greater mechanical strength, which affects the type 45 composite laminate with higher shear strength. Composite 3 of type 90 had very low shear characteristics in this test. The poor fracture-resistance properties of Raffia fiber and its low density are the major causes of this.

### 3.4. Results of Delamination Test

**Figure 8:**
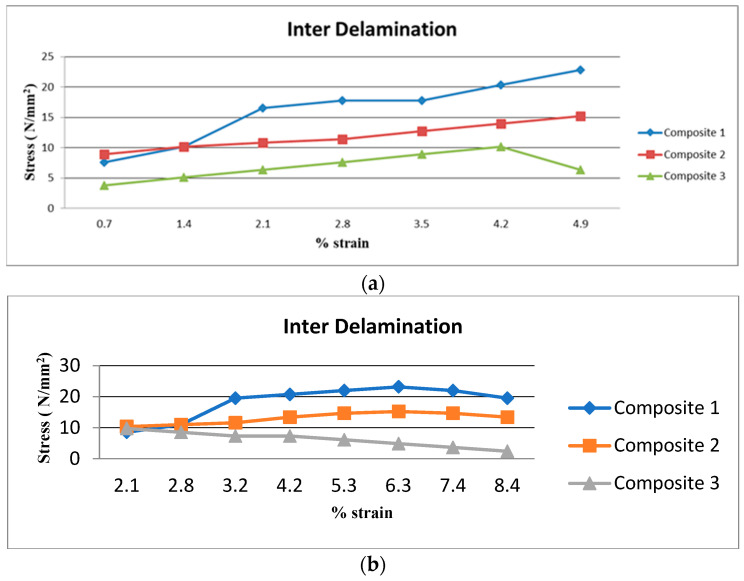

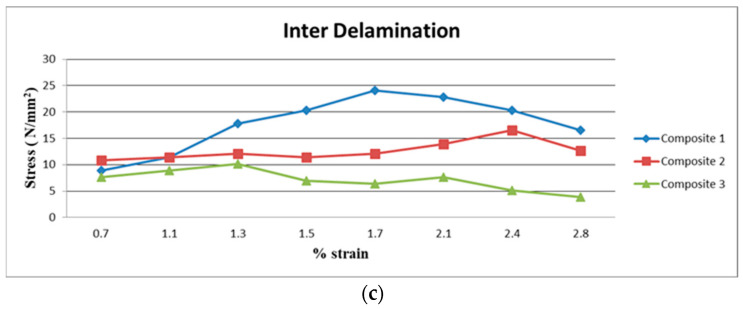
(**a**) Delamination test of 0° orientation in Composites 1, 2 and 3. (**b**) Delamination test of 45° orientation in Composites 1, 2 and 3. (**c**) Delamination test of 90° orientation in Composites 1, 2 and 3.

According to Figure 8a–c, Composite 1 of type 90 exhibits superior interlamination behavior compared to the other composite laminates. The elastic mismatch of neighboring plies, which often results in delamination failure, is removed in this type 90. The stress concentration at the nodal location of intersecting fibers, causes the interply delamination in the failure of these composites. Hence, type 45 and type 0 laminates exhibit inferior strength. Additionally, it has been observed that, Composite 3 with type 45 exhibits very poor inter delamination qualities, mostly as a result of the low mechanical properties of raffia fiber as well as the orientation of the fiber.

### 3.5. Results of Impact Test

Composite 1 of type 0 absorbs more energy than the other Composites 2 and 3, as shown in Table 3. More energy is absorbed by every kind of composite laminate. This is because, there are fiber layers present with an orientation of 0 degrees, which absorb more energy due to continuous layer contact and prevent the creation of cracks during rupture. When compared to type 0 laminate, type 45 absorbs more energy.

### 3.6. Results of Hardness Test

In this study, the Rockwell hardness tester is used to measure the hardness of the Composites 1, 2, and 3. In comparison to other composites, the result demonstrates that Composite 1 of type 45 has a greater degree of hardness. This is mostly due to the acacia fibers’ high strength and density as well as their fiber orientation, which resists indentation in comparison to other composites. It is followed by type 90 composite laminate, and Composite 2 with type 0 exhibits high hardness. Table 4 displays the average value in RHN as well as the three trailing values for the hardness of three different composites.

## 4. Failure Morphological Analysis

In order to examine the internal structural defects in the sample, morphological analysis was performed on tested samples of fabricated composite. In this paper, samples were tested for tensile, flexural, impact, and double shear for morphological analysis, and results are presented.

**Figure 9:**
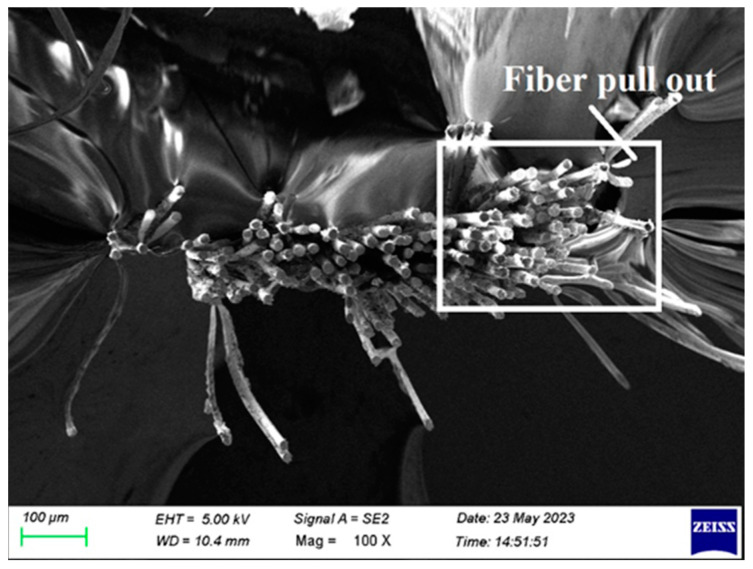
SEM image of Composite 1 of type 45 after the tensile test (Fiber pull out).

Figure 9 shows that, during the tensile test, the fibers pull out. It is mostly the result of incorrect fiber loading and packing during curing. By properly loading the manufactured composite laminate during curing, it is prevented.

**Figure 10:**
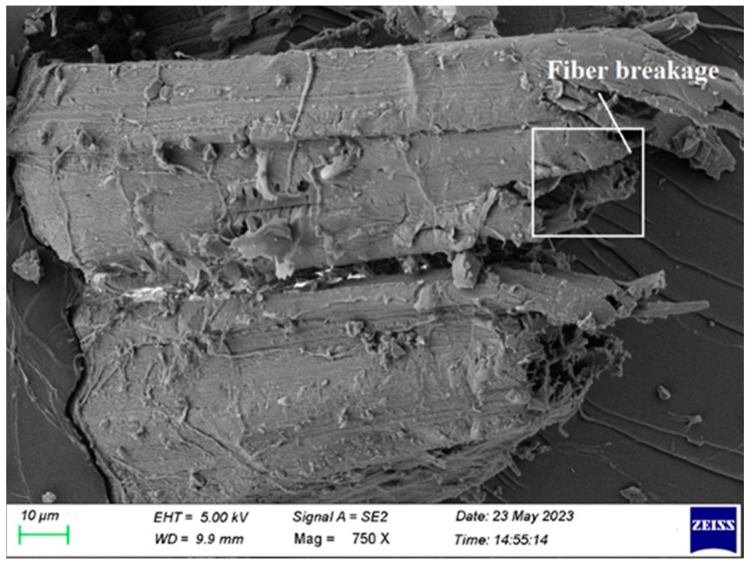
SEM image of Composite 1 of type 45 after the tensile test (Fiber breakage).

Fiber break is shown in Figure 10. By choosing high-quality fiber, break is prevented by utilizing fiber that hasn’t been damaged. The fiber quickly fails, when a composite laminate is loaded, during testing, with damaged fiber. Additionally, morphological examination of fiber is performed to prevent the failure mentioned above.

**Figure 11:**
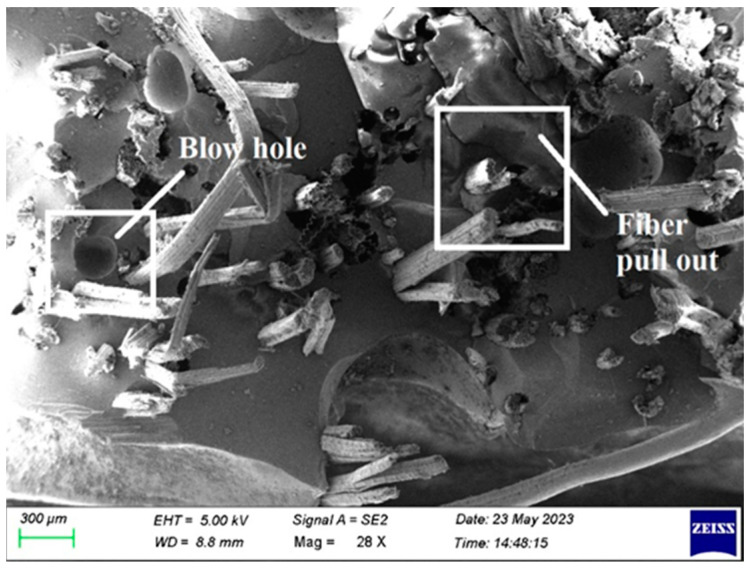
SEM image of Composite 1 of type 90 after impact testing.

Figure 11 shows that, there are blowholes between the fiber layers. Typically, this blow hole reduces the load-bearing capacity of laminate fabrication and weakens the laminate strength. By appropriately placing the glue between each fiber layer, a blowhole is prevented. During the fabrication of composite laminate, the hardener and resin are mixed properly before application.

**Figure 12:**
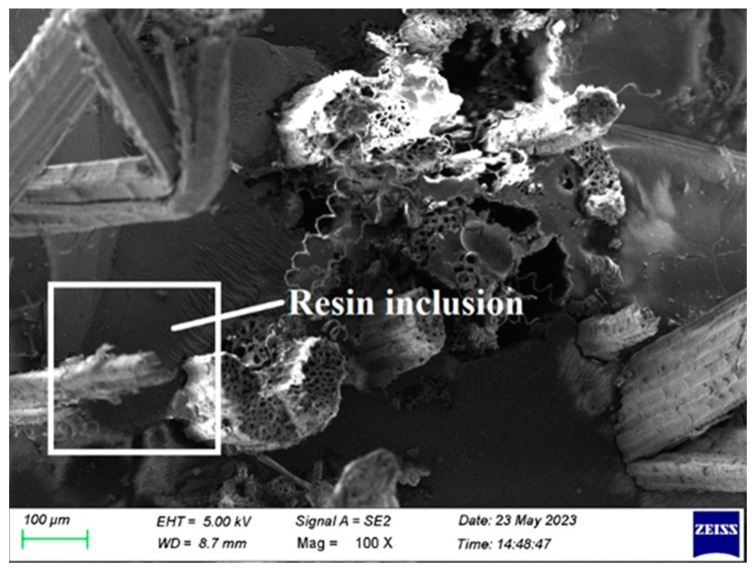
SEM picture of the composite 2 of type 45 after the tensile test.

**Figure 13:**
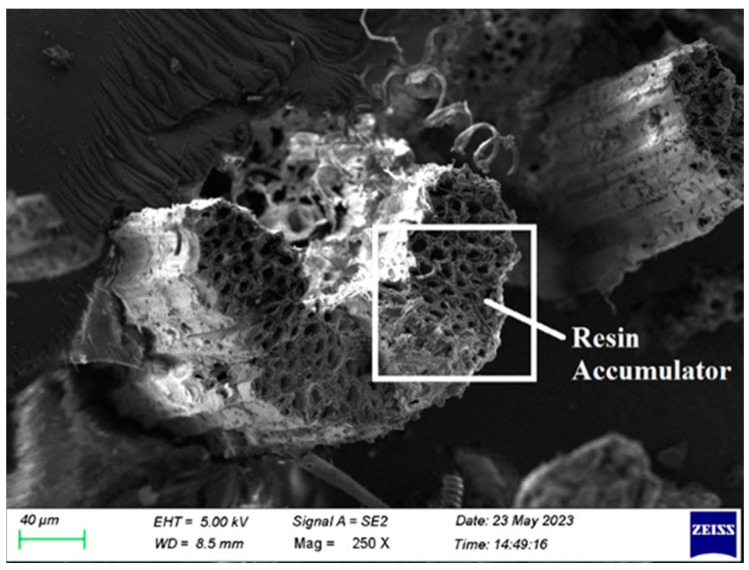
SEM picture of the Composite 2 of type 45 after the tensile test.

Figure 12 and Figure 13 show resin inclusion, which is a result of insufficient resin hardener blending between the fiber layers and inappropriate spreading. It is also a result of the hardener and resin being mixed incorrectly. It is prevented by properly combining the glue and hardener and allowing enough time for curing.

**Figure 14:**
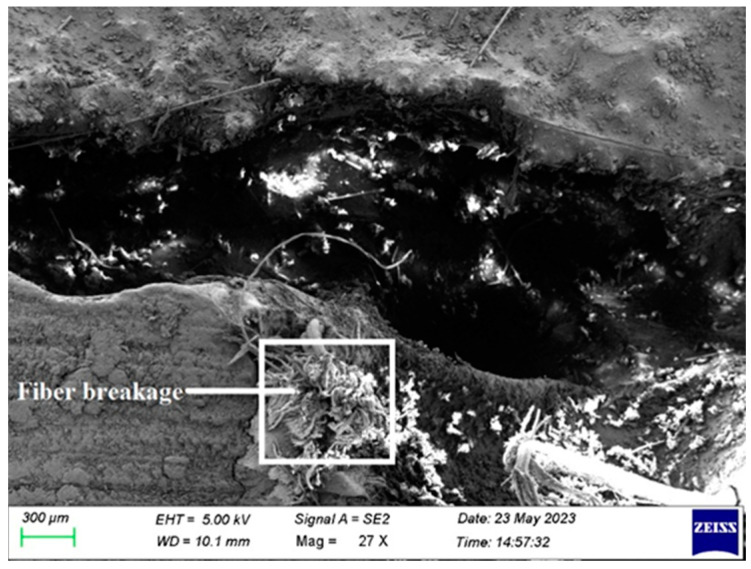
SEM image of Composite 1 of type 45 after flexural testing (Fiber breakage).

Figure 14 also shows fiber breakdown during the flexural test, similar to Figure 10. This is prevented by choosing and using fibers properly before creating a fiber laminate.

**Figure 15:**
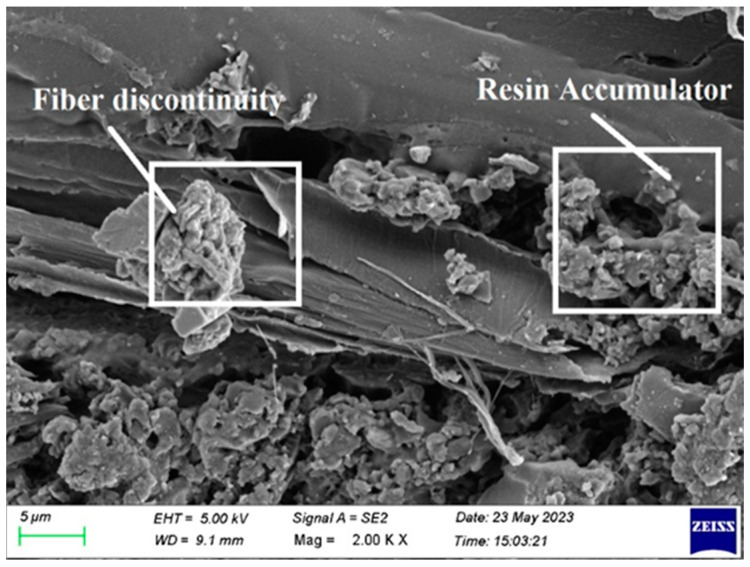
SEM image of Composite 1 of type 45 after flexural testing.

Resin buildup and fiber discontinuity are shown in Figure 15. Fiber discontinuity occurs when testing with short fibers. Consequently, utilizing long fibers while creating composite laminate is advised.

**Figure 16:**
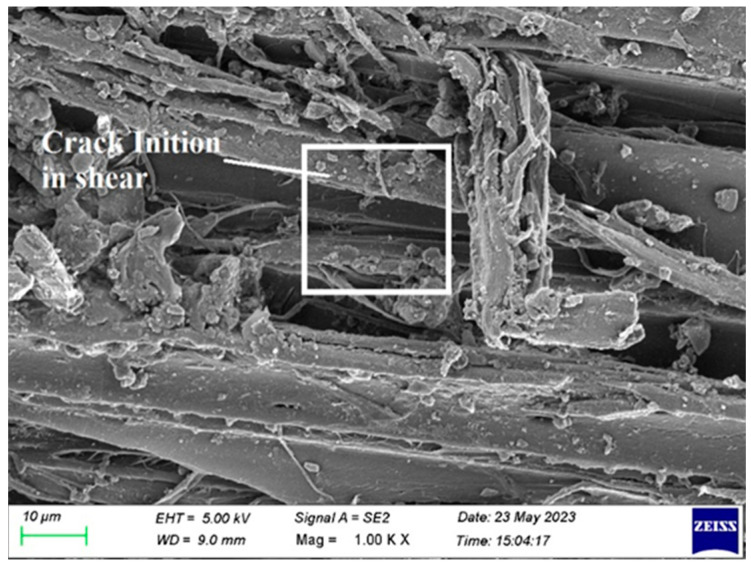
SEM image of Composite 1 of type 45 after shear testing.

Figure 16 shows a fracture initiation in the center of the laminate, which is made up of several layers. The variation in the amount of acacia and raffia fibers causes a split to appear in the center of the laminate, because the fiber volume varies from layer to layer. It is avoided by utilizing the appropriate amount of fiber in each layer for the kind of laminate.

**Figure 17:**
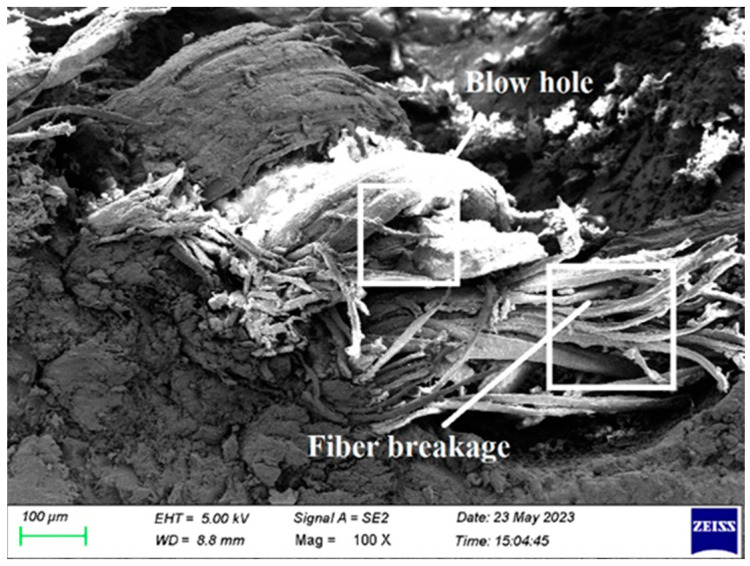
SEM picture of the Composite 1 of type 45 after the double shear test (Blow hole, Fiber breakage).

**Figure 18:**
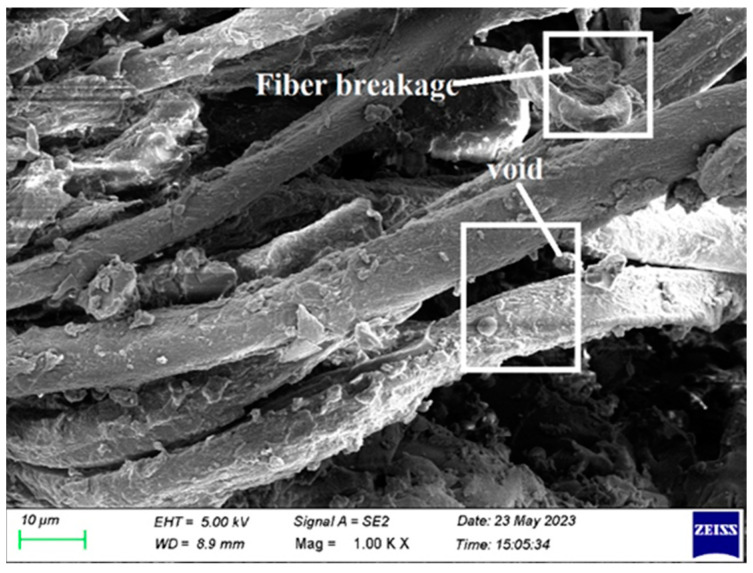
SEM picture of the Composite 1 of type 45 after the double shear test.

Fiber breakage and blow holes are seen in Figure 17 and Figure 18. The selection of low-quality fiber and the inappropriate application of the resin-hardener combination to the fiber are the major causes. It is prevented, by using high-quality fiber and using resin hardener mixtures that are properly mixed and applied.

**Figure 19:**
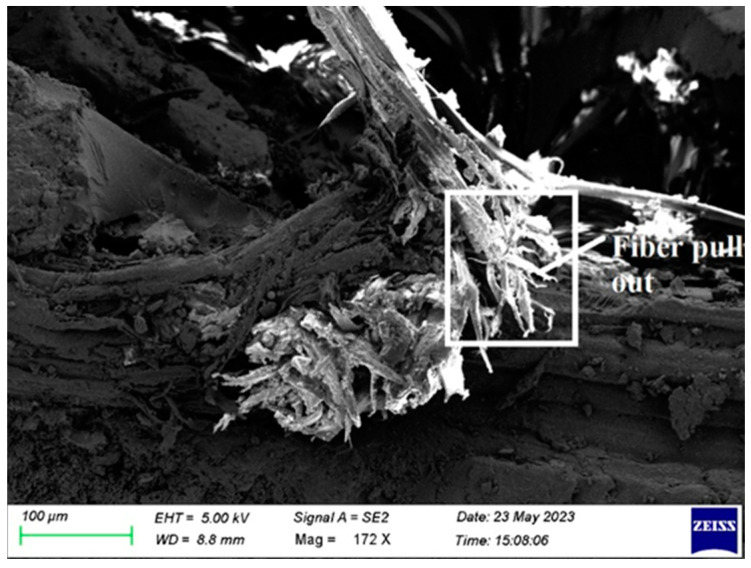
SEM picture of the Composite 3 of type 45 after the flexural testing.

Again, fiber withdrawal is shown in Figure 19. It results from improper laminate loading during cure. As a result, it is advised that the laminate has to be loaded properly during the composite curing.

## 5. Conclusions

In this work, the mechanical and morphological properties are investigated through experiments and calculations, and the following conclusions are listed:

1. Tensile Strength shows that, Composite 1 with type 45 fiber orientation has a very high tensile strength. This is because, acacia fiber has a high tensile strength, because the fibers are aligned at a 45° angle and lock into each other.
2. Flexural Strength shows that, Composite 1 with type 45 fiber orientation laminate has a high flexural strength and is followed by Composite 2 with a type 45 fiber orientation laminate.
3. The double shear test result shows that, the current mechanical behaviors, namely tensile and flexural strength are comparable to earlier mechanical behaviors. Comparing Composite 1 of type 45 to other samples, it exhibits superior double shear behavior.
4. The Composite 1 of type 90 exhibits superior interlamination behavior than other composite laminates.
5. The Composite 1 of type 0 absorbs more energy than the other Composites 2 and 3.
6. In comparison with other composites, the results demonstrates that, Composite 1 of type 45 has a greater degree of hardness.
7. Morphological failure analysis has been carried out to find the few defects. These defects are avoided by proper loading of the fiber, selection of the fiber, and packing during curing.

## Figures and Tables

**Table 1:** Properties of Acacia and Raffia Fibers.

Name of the Fiber	Fiber Density (g/cm^3^)	Tensile Strength in Tensile Test (MPa)	Specific Young’s Modulus in Tensile Test (GPa/g/cm^3^)	Young’s Modulus in Bending Test (GPa)
Raffia Fiber	0.11–0.35	11.7–44.5	2.25–29.37	1.02–4.20
Acacia Fiber	0.906	71.63	84.76	4.21

**Table 2:** Types of composite laminate fabricated.

Type of Composite	Fiber Orientation	Sample Name
Composite 1- Acacia fiber	0°	Type 0
	45°	Type 45
	90°	Type 90
Composite 2- Acacia fiber + Raffia fiber	0°	Type 0
	45°	Type 45
	90°	Type 90
Composite 3- Raffia fiber	0°	Type 0
	45°	Type 45
	90°	Type 90

**Table 3:** Results of Impact Test.

Composite Type	Fiber Orientation	Trial 1	Trial 2	Trial 3	Average Value of Energy Absorbed (J)
Composite 1- Acacia fiber	0°	10	11	13	11.3
	45°	9	10	10	9.6
	90°	10	11	11	10.6
Composite 2- Acacia fiber + Raffia fiber	0°	10	12	11	11
	45°	10	9	9	9.3
	90°	11	9	10	10
Composite 3- Raffia fiber	0°	11	11	10	10.6
	45°	10	10	9	9.6
	90°	11	10	10	10.3

**Table 4:** Results of Hardness Test.

Composite Type	Fiber Orientation	Trial 1	Trial 2	Trial 3	Average Value of Hardness (RHN)
Composite 1- Acacia fiber	0°	38	37	37	37.3
	45°	44	42	43	43
	90°	35	33	35	34.3
Composite 2- Acacia fiber + Raffia fiber	0°	36	38	38	37.3
	45°	43	42	42	42.3
	90°	34	34	35	34.3
Composite 3- Raffia fiber	0°	35	35	37	35.6
	45°	41	42	44	42.3
	90°	32	33	32	32.3

## Data Availability

Not appliable.

## References

[1] Kumar A.L., Prakash M. (2021). The effect of fiber orientation on mechanical properties and machinability of GFRP composites by end milling using cutting force analysis. Polym. Polym. Compos..

[2] Dawit J.B., Regassa Y., Lemu H.G. (2019). Property Characterization of Acacia Tortilis for Natural Fiber Reinforced Polymer Composite. Results Mater..

[3] Arthanarieswaran V.P., Kumaravel A., Saravanakumar S.S. (2015). Characterization of New Natural Cellulosic Fiber from Acacia leucophloea Bark. Int. J. Polym. Anal. Charact..

[4] Chen Y., Su N., Zhang K., Zhu S., Zhu Z., Qin W., Yang Y., Shi Y., Fan S., Wang Z. (2018). Effect of fiber surface treatment on structure, moisture absorption and mechanical properties of luffa sponge fiber bundles. Elsevier Ind. Crops Prod..

[5] Sathish P., Kesavan R., Vijaya Ramnath B. (2015). Experimental investigation on flexural property of abaca and raffia hybrid composites. ARPN J. Eng. Appl. Sci..

[6] Vinod A., Sanjay M.R., Suchart S., Jyotishkumar P. (2020). Renewable and sustainable biobased materials: An assessment on biofibers, biofilms, biopolymers and biocomposites. J. Clean. Prod..

[7] Elenga R.G., Dirras G.F., GomaManiongui J., Djemia P., Biget M.P. (2009). On the microstructure and physical properties of untreated raffia textilis fiber. Elsevier Compos. Part A Appl. Sci. Manuf..

[8] Gabr S., Nikles S., Wenzig E.M.P., Ardjomand-Woelkart K., Hathout R.M., El-Ahmady S., Motaal A.A., Singab A., Bauer R. (2018). Characterization and optimization of phenolics extracts from Acacia species in relevance to their ant inflammatory activity. Biochem. Syst. Ecol..

[9] Kaliappan P., Kesavan R., Vijaya Ramnath B. (2017). Investigation on effect of Fiber hybridization and orientation on mechanical behaviour of natural Fiber epoxy composite. Bull. Mater. Sci..

[10] Ouarhim W., Essabir H., Bensalah M.-O., Zari N., Bouhfid R. (2018). Structural laminated hybrid composites based on raffia and glass fibers: Effect of alkali treatment, mechanical and thermal properties. Elsevier Compos. Part B Eng..

[11] Vadivel K.S., Govindasamy P. (2021). Mechanical and water absorption properties of Acacia Arabica bark fiber/polyester composites: Effect of alkali treatment and fiber volume fraction. Mater. Today Proc..

[12] Senthamaraikannan P., Saravanakumar S.S., Sanjay M.R. (2019). Physico-Chemical and Thermal Properties of Untreated and Treated Acacia planifrons Bark Fibers for Composite Reinforcement. Mater. Lett..

[13] SikameTagne N.R., Ndapeu D., Nkemaja D., Tchemou G. (2018). Study of the viscoelastic behaviour of the Raffia vinifera Fibers. Ind. Crops Prod..

[14] de Filho E.G., da Luz F.S., Fujiyama R.T., da Silva A.C.R., Candido V.S., Monteiro S.N. (2020). Effect of Chemical Treatment and Length of Raffia Fiber (Raphia vinifera) on Mechanical Stiffening of Polyester Composites. Polymers.

[15] Fadele O., Oguocha I.N.A., Odeshi A., Soleimani M., Karunakaran C. (2018). Characterization of raffia palm fiber for use in polymer composites. J. Wood Sci..

[16] Sheeba K.J., Alagarasan J.K., Dharmaraja J., Kavitha S.A., Shobana S., Arvindnarayan S., Vadivel M., Lee M., Retnam K.P. (2023). Physico–chemical and extraction properties on alkali–treated Acacia pennata fiber. Environ. Res..

[17] Santhosh N., Selvam S., Reghu R., Sundaran J., Mathew B.C., Palanisamy S. (2023). Mechanical properties studies on rubber composites reinforced with Acacia Caesia fibre. Mater. Today.

[18] Kanimozhi T., Sundara Pandian S., Sathish S. (2022). Influence of Acacia concinna and Vachellia nilotica seed nanopowder on the properties of short Turkish hemp–reinforced epoxy composites. Sage J..

[19] Palanisamy S., Kalimuthu M., Palaniappan M., Alavudeen A., Rajini N., Santulli C., Mohammad F., Al-Lohedan H. (2021). Characterization of Acacia caesia Bark Fibers (ACBFs). J. Nat. Fibers.

[20] Kumar S., Prasad L., Patel V.K., Kumar V., Kumar A., Yadav A., Winczek J. (2021). Physical and Mechanical Properties of Natural Leaf Fiber-Reinforced Epoxy Polyester Composites. Polymers.

[21] Verma D., Goh K.L. (2021). Effect of Mercerization/Alkali Surface Treatment of Natural Fibres and Their Utilization in Polymer Composites: Mechanical and Morphological Studies. J. Compos. Sci..

[22] Mohd Bakhori S.N., Hassan M.Z., Mohd Bakhori N., Jamaludin K.R., Ramlie F., Md Daud M.Y., Abdul Aziz S. (2022). Physical, Mechanical and Perforation Resistance of Natural-Synthetic Fiber Interply Laminate Hybrid Composites. Polymers.

